# Reclassification of the *GRIA3* splice-site variant in an X-linked family with intellectual disability and psychiatric symptoms

**DOI:** 10.3389/fgene.2026.1914453

**Published:** 2026-08-14

**Authors:** Lina Hu, Yuqiong Chai, Xiaofei Liu, Yanan Wang, Hongwei Jiang

**Affiliations:** 1 Medical Genetics/Prenatal Diagnostic Department, Luoyang Maternal and Child Health Hospital, Luoyang, China; 2 The First Affiliated Hospital, and College of Clinical Medicine of Henan University of Science and Technology, Luoyang, China

**Keywords:** AMPAR, GRIA3, intellectual disability, psychiatric symptoms, x-linked

## Abstract

**Background:**

The *GRIA3* gene is located on the X chromosome and encodes a subunit (GluR3) of the a-amino-3- hydroxy-5-methylisoxazole-4-propionic acid receptor (AMPAR). The pathogenic variants of *GRIA3* are mostly associated with neurodevelopmental disorders. Patients were overwhelmingly male and presented mainly with intellectual disability, dystonia, epilepsy and other symptoms.

**Methods:**

In this study, we reported a pedigree that carried a novel splicing site variant of *GRIA3* (c.268 + 1G>C) by whole exome sequencing (WES) and co-segregation analysis. Three affected family members (two males and one female) not only showed intellectual disability but also presented significant psychiatric symptoms and spatial memory deficits. The minigene assay further confirmed that this variant lead to exon 2 skipping.

**Results:**

According to ACMG guidelines, We reclassified previously variant of unknown significance (VUS) into “likely pathogenic” through co-segregates analysis and minigene assay.

**Conclusion:**

It is worth noting that, unlike previous reports, our patients mainly manifested as intellectual disability combined with psychiatric symptoms, expanding the known phenotypic spectrum of GRIA3 gene. Moreover, this variant is the first reported, enriching the database and providing additional evidence to support genetic counselling and prenatal diagnosis.

## 1 Introduction

The *GRIA3* gene encodes the GluR3 subunit of the a-amino-3- hydroxy-5-methylisoxazole-4-propionic acid receptor (AMPAR), located at Xq25. It is associated with a Wu-type X-linked syndromic intellectual developmental disorder (MRXSW; OMIM #300699), showing X-linked recessive (XLR) inheritance. Primary phenotypes include neurodevelopmental disorders (e.g., intellectual disability, autism spectrum disorder), dysmorphic features (e.g., prominent supraorbital ridges, deep-set eyes), movement disorders, epilepsy, and psychiatric conditions. Endogenous AMPARs are tetrameric hetero-oligomers assembled from variable combinations of four subunits, GluR1–GluR4, primarily as GluR1/GluR2 or GluR2/GluR3 heterodimeric complexes (Wenthold et al., 1996). In brain, AMPARs are tethered to postsynaptic membranes and mediate excitatory synaptic transmission. AMPARs also play roles in synaptic plasticity and learning and memory (Bredt and Nicoll, 2003; Chater and Goda, 2014). Mutations in *GRIA3* gene altering channel properties are associated with moderate cognitive impairment of humans (Wu et al., 2007). According to the HGMD database, 61 variants have been reported, primarily involving duplications, missense mutations, and frameshift mutations. Most affected males are inherited from phenotypically normal mothers who are carriers (Sun et al., 2021; Trivisano et al., 2020). Female cases have also been reported, which may be related to X inactivation, epigenetic modifications, or other mechanisms (Okano et al., 2023; Martinez-Esteve Melnikova et al., 2022), yet the exact reasons remain unclear.

Here, we report a pedigree with three patients. They are primarily characterized by ID and psychiatric symptoms, without seizures or abnormal muscle tone. Whole-exome sequencing (WES) detected only one candidate variant (c.268 + 1G>C) in the *GRIA3* gene. To determine the pathogenicity of this site, we first performed Sanger sequencing on family members, confirming the co-segregation of the variant with the disease. And then, Minigene assay was performed to uncover its effects on splicing. Moreover, we discuss and summarize the pathogenic patterns of *GRIA3* gene variants, aiming to provide a theoretical basis for early diagnosis and precise intervention in diseases associated with this gene.

## 2 Materials and methods

### 2.1 Pedigree and clinical evaluation

**FIGURE 1:**
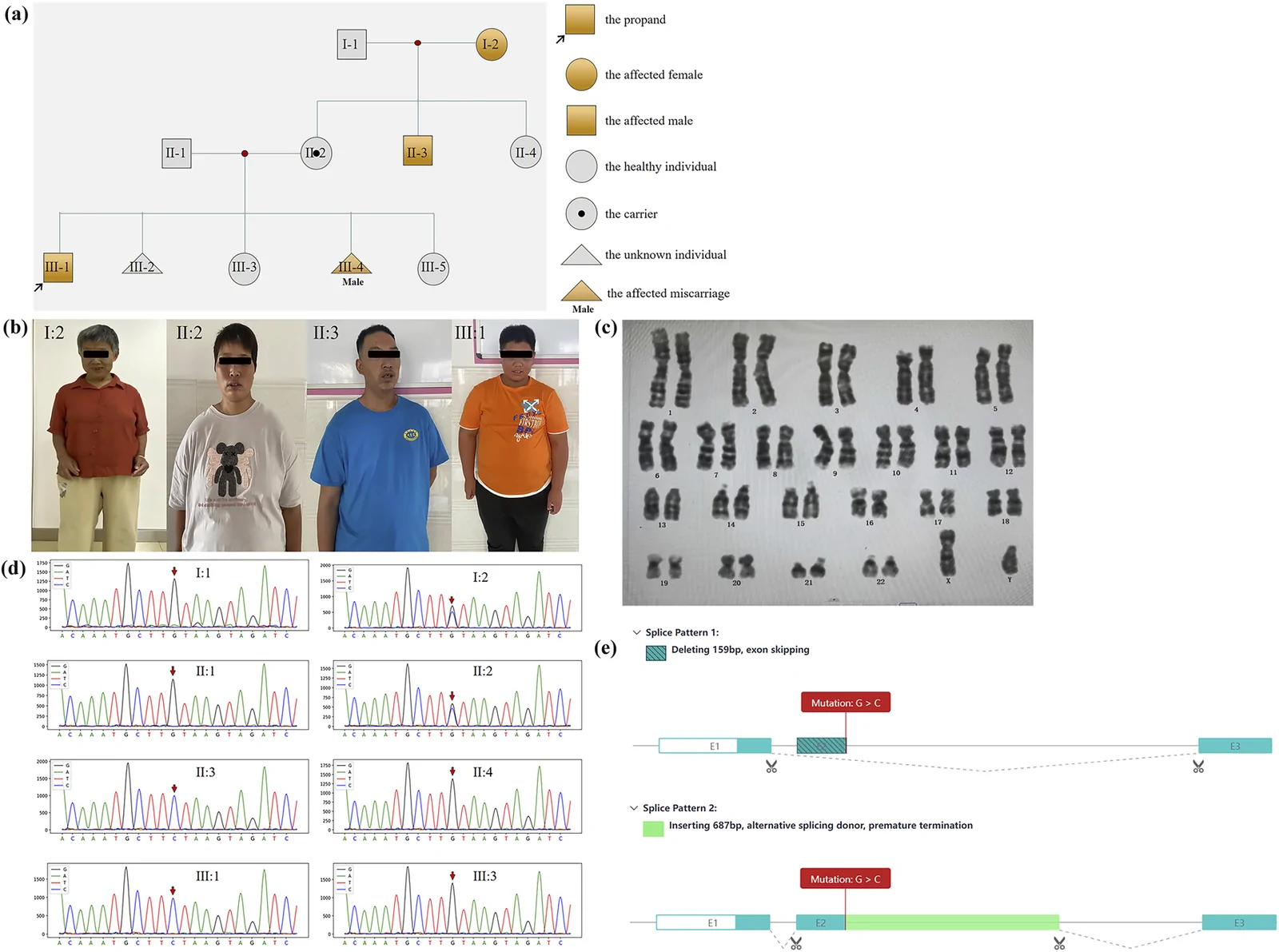
Clinical and genetic findings: **(a)** Pedigree chart of the family (three generations). **(b)** The physical characteristics of the affected individuals. **(c)** Peripheral blood karyotyping analyses of the proband revealed a normal 46, XY karyotype. **(d)** I:2 and II:2 carried heterozygous mutation at the chromosome location chrX:122319843. I:1, II:1,II:4 and III:3 is wild type at chromosome position chrX:122319843. II:3 and III:1 is a hemizygous mutation at the chromosome location chrX:122319843. **(e)** The online RNA Splicer tool predicted two splice patterns, pattern 1 with exon 2 (159bp) skipping and pattern 2 with 687 bp inserting.

This study involved a Chinese family of nine members who presented to the Department of Medical Genetics and Prenatal Diagnosis at Luoyang Maternal and Child Health Hospital. There were three affected individuals: the proband (III-1), his maternal uncle (II-3), and his maternal grandmother (I-2). This study was approved by the Ethics Committee of Luoyang Maternal and Child Health Hospital (Approval No.: LYFY-YCCZ-2025003) and all patients provided verbal and written informed consent. Medical histories were collected for all members, including intellectual assessment, growth and developmental status, age at first onset, medication use, imaging data, and genetic testing results. The family pedigree is depicted in Figure 1a.

### 2.2 Genetic testing

Chromosome karyotyping is performed by G-banding technology in accordance with the International System for Human Cytogenetic Nomenclature (ISCN). The WES based on next-generation sequencing technology was performed for variant detection. Peripheral blood samples are taken from participants, and genomic DNA is extracted using a magnetic bead-based blood genomic DNA extraction kit and a automated extractor (Tiangen, Beijing, China). DNA library preparation was performed using the KAPA HyperPlus Kits (Roche, Switzerland), followed by exome capture with the IDT xGen Exome Research Panel v1.0 (IDT, Coralville, United States). An appropriate amount of genomic DNA was fragmented to 250–300bp peak size using KAPA frag enzyme. Then, the fragmented DNA underwent end repair, A-tailing, ligation with index adapters, and PCR amplification according to manufacturer instructions. The library concentration was detected using a Qubit™ dsDNA HS Assay Kit and Qubit4.0 (Thermo Fisher, USA). The fragment lengths were determined by a QSeq400 fragment analyzer. Next, sequencing was performed on an Illumina NovaSeq 6,000 platform in PE150 mode. Approximately 10G of data were generated, achieving a Mean exome sequencing depth 100X, 90.00% of the target region sequencing depth >20X coverage. Finally, the reads were aligned against the reference genome (GRCh37/hg19) using Burrows-Wheeler Aligner (BWA, version: 0.59) software. Base quality scores were re-calibrated using the Genome Analysis Toolkit (GATK version 4.2.0), the single nucleotide variations (SNVs) and insertions/deletions (indels) were detected and filtering. Variants were annotated using the VEP software based on the Refseq annotation of human (hg19) downloaded from UCSC database. The copy number variations (CNVs) is called with CNVkit (version 0.9.9) and annotated by annots (version 0.9.9). For the autosomal recessive and X-linked model, the variants were filtered for a minor allele frequency (MAF) < 0.01, with the MAF threshold for dominant disorders set to 0.001. Variant validation and co-segregation analysis were confirmed by polymerase chain reaction (PCR) and Sanger sequencing.

### 2.3 Construction of minigene

To assess the splicing effects of the variant (*GRIA3*:c.268 + 1G>C), we first performed bioinformatics prediction of its potential impact using SpliceAi and the RDDC software (https://rddc.tsinghua-gd.org/). Subsequently, For the minigene assay, vector pSPL3, referred to as the exon trapping vector, was selected as the experimental system. Which encodes two vector exons (SD6 and SD2) containing functional splicing sites. And a homologous recombination cloning strategy was adopted to construct recombinant plasmids, using the One-Step Seamless Cloning Kit (SC612) (Genesand Bio, Beijing, China). In brief, First, the fragments encompassing the target *GRIA3* mutation exon and partial upstream/downstream intron sequences were amplified with specific primer pairs (TY-GRIA3-F/TY-GRIA3-R) from genomic DNA. PCR products were recovered by using purification kits (A0712B, Tian Gen, Beijing, China) for subsequent use. Then, amplified fragments were digested and cloned into the NdeI and XhoI sites of pSPL3 to obtain the wild-type (WT) plasmid. Meanwhile, the recombinant plasmid was transformed into *E. coli* DH5α competent cells and cultured overnight at 37 °C in LB medium containing 100 μg/mL ampicillin. Positive transformants were obtained by screening for ampicillin-resistant colonies. Endotoxin-free plasmids were prepared using an endotoxin-free plasmid kit (Tian gen, Beijing, China), and sequence correctness was verified by Sanger sequencing. Building upon this foundation, we further constructed *GRIA3* mutant-type (MT) plasmids. To achieve this, two pairs of primers (TY-GRIA3-F/TY-GRIA3-R-Z and TY-GRIA3-F-Z/TY-GRIA3-R) incorporating the target mutation sites were designed. Similarly, PCR amplification was performed using the WT plasmid as a template. Subsequently, site-directed mutagenesis was achieved via with the One-Step Seamless Cloning Kit (SC612) (Genesand Bio, Beijing, China) and homologous recombination method. All primer information is listed in the supplementary materials (Supplementary Table S1).

### 2.4 Cell culture, transfection, and detection

HEK293T cells were from our laboratory stock. HEK293T cells were cultured in Dulbeccos Modified Eagle medium (DMEM) supplemented with 10% fetal bovine serum (FBS), penicillin (100 U/mL), and streptomycin (100 U/mL) at 37 °C in a humidified atmosphere with 5% CO2. On the afternoon of the day prior to the experiment, HEK293T cells were seeded into 12-well plates Lipofectamine 3,000 transfection reagent (Invitrogen, Waltham, MA, USA) following the manufacturer’s instructions. The cells were harvested 48 h post transfection. Total RNA was isolated with Cell Total RNA Isolation Kit (Foregene, Chengdu, China), and cDNA was generated using FAST RT reagent Kit (Takara Bio, Inc., Otsu, Japan). Minigene-derived transcripts were analyzed by RT-PCR, which was performed using pSPL3 vector specific primers (SD6-F/SA2-R). Agarose gel electrophoresis was used to verify transcript size. The aberrantly spliced transcripts were eluted from the gel and sequenced to determine the precise splice modes. All primer information is listed in the supplementary materials (Supplementary Table S1).

## 3 Results

### 3.1 Clinical findings

The proband and his maternal uncle presented with similar clinical manifestations, primarily characterized by severe ID and slightly delayed developmental milestones. However, there are no significant motor impairments in adulthood. The severity of ID is primarily assessed through a comprehensive evaluation of intellectual development and adaptive behavior. The only facial feature potentially noteworthy was a prominent supraorbital ridge and deep-set eyes (Figure 1b). Symptoms became clearly apparent around the age of 6 years with violent tendencies and sleep disturbances. They are both primarily characterized by ID and psychiatric issues. All had a diagnosis of ID with significant behavioral deficits requiring attention or treatment. Their behavioral manifestations may include emotional instability, delusions, hallucinations, and other psychiatric symptoms. Risperidone and clozapine demonstrated good efficacy, while lorazepam effectively improved sleep disturbances. The maternal grandmother of the proband primarily presented with spatial memory defect, reflected in a failure to recall the way home. ID and sleep disturbances were also present, but violent tendencies and other psychiatric symptoms were not prominent.

Physical examination, electroencephalogram (EEG), and electromyogram (EMG) showed no significant abnormalities. Brain MRI of the proband showed reduced volume in both frontal lobes. The maternal uncle’s MRI was normal. Meanwhile, the maternal grandmother’s cranial MRI showed abnormal white matter signals in the periventricular regions of both lateral ventricles, the frontotemporal lobes, and the centrum semiovale (Fazekas 2).

### 3.2 Variant analysis

Chromosomal analysis from peripheral blood samples was performed on III-1 and I-2 using standard G-banding techniques. No abnormality was observed in III-1’s chromosomes (Figure 1c). Unfortunately, chromosomal analysis of I-2 could not be conducted due to failed cell culture, potentially related to her taking antihypertensive medication. For III-1 and I-2, the WES was carried out to detect SNVs, indel, and CNVs. Based on WES data, mitochondrial DNA analysis yielded no candidate variants. After filtering, only one variant in *GRIA3* (c.268 + 1G>C), a gene associated with ID was identified. This variant was absent in gnomAD. According to the American College of Medical Genetics and Genomics (ACMG) criteria (PM2_Supporting and PVS1_Moderate) (Richards et al., 2015; Abou Tayoun et al., 2018), it is classified as a variant of uncertain significance (VUS). We conducted site validation via Sanger sequencing in affected individuals within three generations. It was shown that the proband inherited the variant from his asymptomatic mother, while both his maternal uncle and mother inherited it from his affected maternal grandmother. No other family members were found to carry the variant (Figure 1d). On review of the family history, we speculate that the proband’s maternal grandmother may have had a *de novo* mutation; however, her parents are deceased, precluding verification.

### 3.3 The *GRIA3*: c.268 + 1G>C variant affects splicing

**FIGURE 2:**
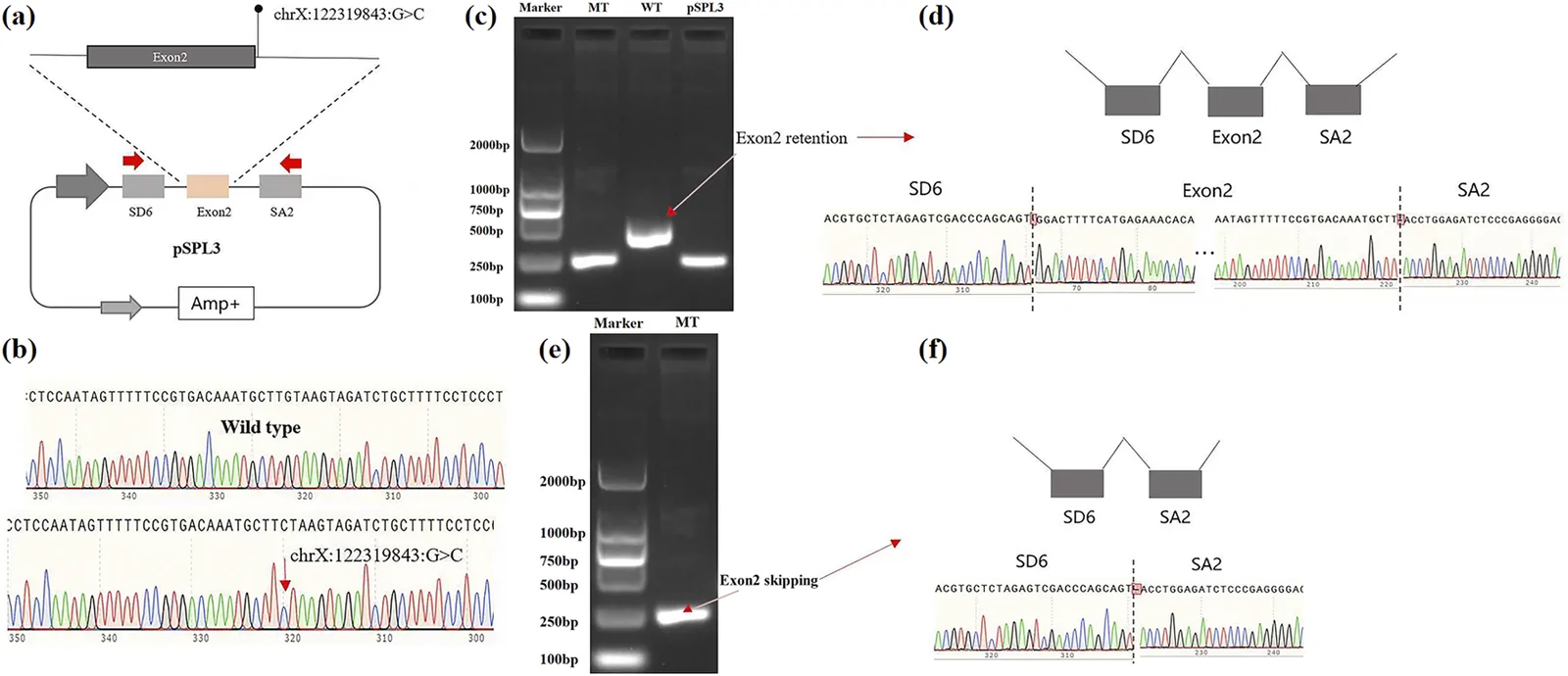
Results of the *GRIA3:* c.268 + 1G>C variant *in vitro* minigene functional studies. **(a)** Schematic diagram of the minigene and vector construction. **(b)** Sequencing in the recombinant vector. The top of Figure **(b)** indicates the results of wild-type (WT) minigene sequencing, and the bottom shows the sequencing of the MT (mutant-type) (c.268 + 1G>C) minigene. Both are partial sequencing results. The red arrows indicate mutated positions in the Sanger sequencing results. **(c,d)** Agarose gel electrophoresis and Sequencing analysis of RT-PCR products. The RT-PCR product derived from HEK293T cells transfected with a plasmid construct harboring the WT gene. The pSPL3 group represents the “empty” vector transfected group. **(e,f)** Agarose gel electrophoresis and Sequencing analysis of RT-PCR products. The RT-PCR product derived from HEK293T cells transfected with a plasmid construct harboring the MT gene.

The SpliceAi predicted that a novel splicing variant (*GRIA3*:c.268 + 1G>C) may induce exon 2 skipping (159bp). An online RNA splicing tool (RDDC RNA splicer) predicted two possible abnormal splicing pattern: exon 2 skipping (159bp) and insertion of 687 bp (Figure 1e). To analyze how the *GRIA3*:c.268 + 1G>C variant affects splicing, both WT and MT *GRIA3* minigenes encompassing exon 2 and adjacent intron sequences were constructed (Figure 2a). The successful construction of recombinant plasmids were confirmed by Sanger sequencing (Figure 2b). The minigenes were transfected into HEK293T cells, and splicing patterns were analyzed. The WT minigene transfected into HEK293T cells yielded a single 422bp product (Figure 2c). By sequencing, it was confirmed that the 422bp product represented correct splicing of exon 2 (Figure 2d). The MT minigene transfected into HEK293T cells yielded a 263bp product due to exon 2 skipping (Figures 2e,f). No additional PCR products were observed in the MT minigene group. Thus, only one splice variant—exon 2 skipping—was generated after introducing the *GRIA3*:c.268 + 1G>C variant into HEK293T cells.

### 3.4 Summary of pathogenicity evidences

The *GRIA3*:c.268 + 1G>C variant is not present in databases of normal controls. PM2_Supporting evidence is available. It was located in the canonical splice region of the second intron of GRIA3 gene at the position −1 of the donor splice site and may result in impaired gene function. The length of the missing exon is less than 10% of the protein length. PVS1_Moderate is available. To confirm the pathogenicity of this variant, we first performed a co-segregation analysis in the three-generation pedigree. Among them, II-3 and III-1 are hemizygous and both are patients. I-2 and II-2 are carriers. I-2 is also a patient. I-1, II-1, II-4, III-3, and III-5 are all wild-type (Figure 1a). III-1, II-3 and I-2 are all affected individuals who present with ID and spatial memory impairment. In addition, III-1 and II-3 exhibit violent tendencies, emotional instability, delusions, and hallucinations. Family members who do not carry this variant are all unaffected. It revealed co-segregation of the phenotype with the heterozygous state of the variant. PP1_Moderate is available. A minigene splicing assay further validate that it might cause exon 2 skipping. PVS1_Strength (RNA) is available (Walker et al., 2023).

## 4 Discussion

This study reports a family with three affected individuals, primarily presenting with ID, psychiatric symptoms, and spatial memory deficits. Language and motor milestones were delayed compared to typical development, but no significant muscle tone issues were observed in adulthood. Genetic testing identified a variant (*GRIA3*:c.268 + 1G>C), associated with developmental intellectual disability syndrome, Wu type, exhibiting XLR inheritance. We performed the co-segregation analyses and minigene assay, supporting pathogenicity of this variant and implicating it as the most likely causing gene in this family.

Currently, the most reported GRIA3 variants are associated with neurodevelopmental disorders (NDDs); Both loss-of-function (LoF) and gain-of-function (GoF) variants could be pathogenic in either sex. The clinical manifestations and severity vary based on the variant type (LOF or GOF) and location (Rinaldi et al., 2024). The GoF variants exhibit more severe clinical phenotypes, including earlier onset of seizures, increased muscle tone, and more frequent dyskinesias. In comparison, The patients with LoF variants show later-onset epilepsy (median age: 16 months) and predominantly present with hypotonia and sleep disorders (Rinaldi et al., 2024). All three affected individuals suffered from sleep disturbances. The GRIA3 polymorphism that was previously found to be associated with depressive disorder in women showed an association with sleep duration in healthy women (Utge et al., 2011). Nevertheless, sleep disturbances were observed in both males and females in our cases. Therefore, we suggest that it may be related to the variant itself and its impact on protein function. In 1999, the GRIA3 gene was identified as a candidate gene for bipolar disorder and nonspecific X-linked ID (Gécz et al., 1999). The degree of ID in the affected family members ranged from mild to severe (Trivisano et al., 2020), manifesting from infancy with delayed psychomotor development accompanied by severe language acquisition lags and impairments (Jin et al., 2015). In this study, all three patients suffered from moderate to severe ID, self-care in life, poor reading and numeracy skills, and poor interpersonal skills. None of the three patients developed epilepsy, abnormal muscle tone, or movement disorders in adulthood. However, as recalled by the proband’s parents, the proband exhibited delayed milestones in head control, walking, and language acquisition compared to peers.

**FIGURE 3:**
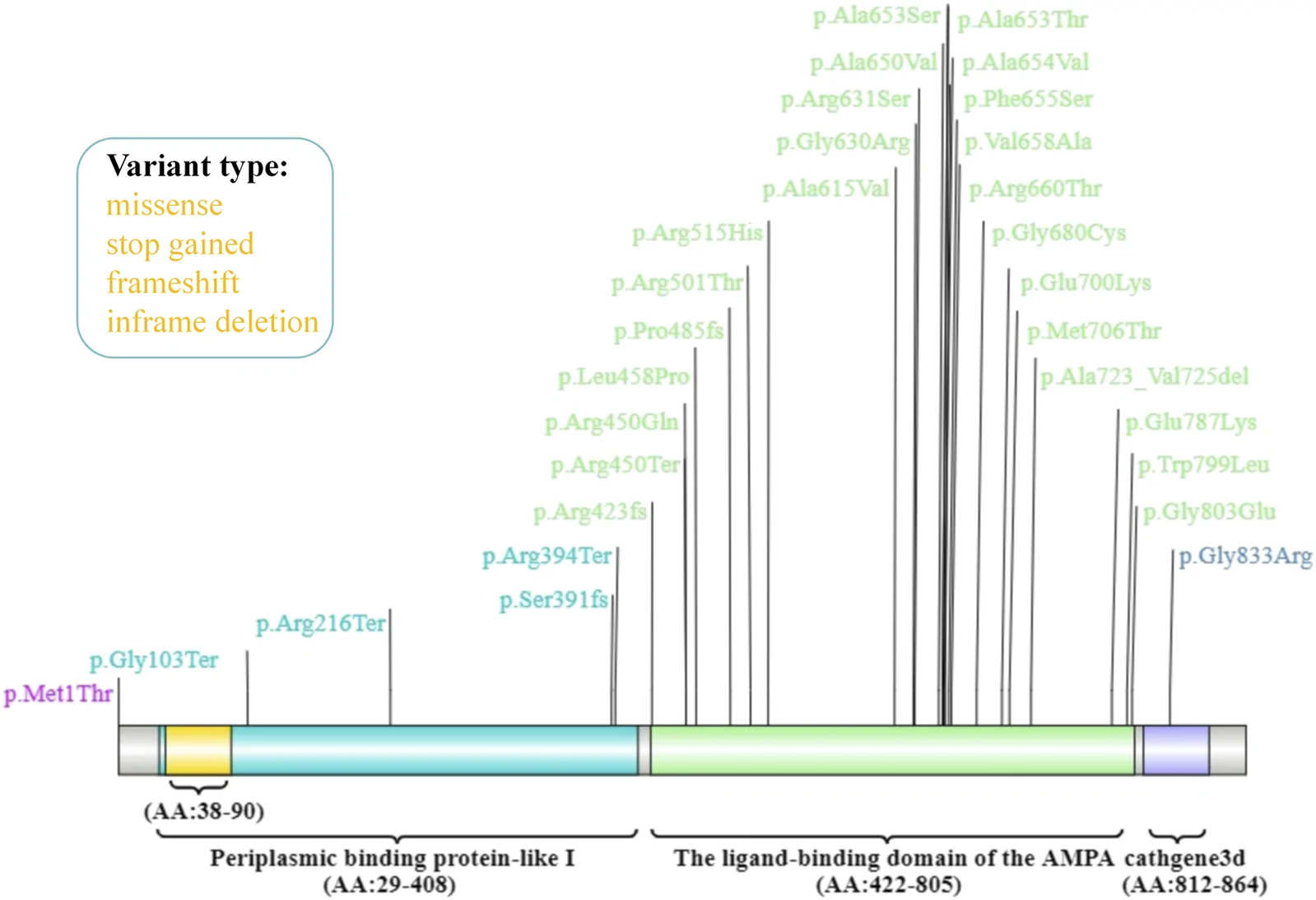
Distribution of all pathogenic and likely pathogenic variant in *GRIA3* gene. It is as annotated on Interpro (https://www.ebi.ac.uk/interpro/protein/UniProt/P42263/), along with the representative structural domains of GluR3 protein and variant type (missense stop gained, frameshift, inframe deletion). The area marked yellow is the position corresponding to exon 2. AA: amino acid positions.

Canonical splice sites in human genes are found at exon-intron junctions, including splice donor and acceptor sites. Mutations here often disrupt pre-mRNA splicing, causing issues like exon skipping and intron retention (Anna and Monika, 2018). The *GRIA3* gene shows very low mRNA levels in blood (TPM: 0.01), making it suitable for splice site mutation analysis via a minigene assay. The minigene experiment confirmed that the mutation (*GRIA3*:c.268 + 1G>C) caused a skipping of exon 2 (159 bp), which may alter gene structure and impairing protein function. The GluR3 protein are composed of mainly two domains, Periplasmic binding protein-like I and The ligand-binding domain of the AMPA. The former initiates dimerization, ensuring that only subunits from specific iGluR families can bind to each other. The latter serves as the subunit binding site and is the site of action (Greger and Esteban, 2007). We also compiled currently reported pathogenic or likely pathogenic sites (Figure 3), revealing that most variants within the second domain, which may relate to its function. Our variant is located in exon 2 (amino acid sequence 38–90), which belongs to the first domain. Deletion of exon 2 may cause abnormal subunit dimerization, thereby impairing AMPAR function. And the protein region of exon 2 of the *GRIA3* gene is highly conserved among different species. In addition, we also noted that the *GRIA3* gene exhibits five distinct splicing patterns in the alternative splicing database, of which exon 2 is an essential component. Therefore, it is believed to be crucial for GRIA3 protein function.

Due to the proband’s ID, his mother desired to have a healthy child and consulted our department. First, we conducted a genetic analysis on the proband. WES only identified the *GRIA3*: c.268 + 1G>C variant. This variant has not been reported in the database and is located at the classical splicing site, which is predicted to affect splicing. According to the guideline of ACMG, PM2_Supporting and PVS1_Moderate (Richards et al., 2015; Abou Tayoun et al., 2018) are available. At this stage, the variant is classified as VUS. Later, through sanger sequencing, it was found that all three patients with the abnormal phenotype carried this variant, while family members without the phenotype did not carry it, demonstrating co-segregation between the variant and the phenotype. The evidence PP1_Moderate was added. To verify that it does affect splicing, we manipulated the minigene assay, and the result showed a skipping of exon 2. Now, we can apply the strong evidence PVS1_Strength (RNA) (Walker et al., 2023). Based on all the above evidence, PVS1_Strength (RNA), PM2_Supporting, and PP1_Moderate, this variant has been reclassified as likely pathogenic. ID is a type of disease that exhibits both clinical and genetic heterogeneity. This requires us to be able to promptly identify and conduct genetic analysis. Early molecular diagnosis may provide an opportunity for intervention, thereby improving the long-term outcomes of the disease.

The limitations of the study lie in the following two aspects. First, the detection of X chromosome inactivation was not conduct in our study. The phenotype of the proband’s grandmother may be related to X chromosome inactivation. However, the *GRIA3* gene is a variable escape gene, and its expression is mainly in brain tissue (Tukiainen et al., 2017). We believe that the X chromosome inactivation detection might not be the most effective method. Crucially, we performed WES on the proband’s grandmother but found no other relevant variants. Second, this variant has not been verified at the protein level, and the electrophysiological changes have not been measured. Which will be the next step work.

## 5 Conclusion

In this study, we described a three-generation chinese family with ID. WES was applied to identify a unreported mutation in *GRIA3* (c.268 + 1G>C), whereas linkage analysis was applied to confirm cosegregation of the mutation and phenotype. The minigene *in vitro* splicing assay demonstrated that this variant affects splicing, leading to exon 2 skipping; In conclusion, our results further support that this variant is likely pathogenic and may be the cause of their phenotype, which also provide evidence for its prenatal diagnosis and genetic counseling.

## Data Availability

The original contributions presented in the study are publicly available. This data can be found in the Genome Sequence Archive for Human (GSA-Human) repository of the National Genomics Data Center, with the accession number HRA019902, at: https://ngdc.cncb.ac.cn/gsa-human/browse/HRA019902.
