# Supplementary material for: Reclassification of the GRIA3 splice-site variant in an X-linked family with intellectual disability and psychiatric symptoms
Source: Front Genet. 2026 Aug 14;17:1914453. doi: 10.3389/fgene.2026.1914453 (PMC13522893; doi:10.3389/fgene.2026.1914453)
Supplement: Supplementary file 1 [file Table1.docx]

**Supplementary Table S1.** Primers used in this study.

| Primer name | Sequence (5´- 3´) | Size of pcr products | |
| --- | --- | --- | --- |
| TY-GRIA3-F | TCACCAGAATTCTGGAGCTCTTGGTTCCATCGTCCTAAC | 682bp | |
| TY-GRIA3-R | TTATGTACCTCTGTATCATGCCCACTGTCTTGAGTTTTTC |  |  |
| TY-GRIA3-F | TCACCAGAATTCTGGAGCTCTTGGTTCCATCGTCCTAAC | 440bp | |
| TY-GRIA3-R-Z | CTACTTAGAAGCATTTGTCACGGAAAAACTATTGGA |  |  |
| TY-GRIA3-F-Z | AATGCTTCTAAGTAGATCTGCTTTTCCTCCCT | 225bp | |
| TY-GRIA3-R | TTATGTACCTCTGTATCATGCCCACTGTCTTGAGTTTTTC |  |  |
| SD6-F | TCTGAGTCACCTGGACAACC | 422bp (WT) | 263bp(MT) |
| SA2-R | ATCTCAGTGGTATTTGTGAGC |  |  |

**WT:** The group was transfected with wild plasmids. **MT:** The group was transfected with mutant-type plasmids.
